# Functional Ingredients From *Brassicaceae* Species: Overview and Perspectives

**DOI:** 10.3390/ijms21061998

**Published:** 2020-03-15

**Authors:** Daniela Ramirez, Angel Abellán-Victorio, Vanesa Beretta, Alejandra Camargo, Diego A. Moreno

**Affiliations:** 1 Laboratorio de Cromatografía para Agroalimentos, Facultad de Ciencias Agrarias, UNCuyo, Mendoza 54 261, Argentina; danielaandrearamirez2511@gmail.com (D.R.); vanesaberetta@hotmail.com (V.B.); alebcamargo@gmail.com (A.C.); 2 Instituto de Biología Agrícola de Mendoza, CONICET Mendoza 54 261, Argentina; 3 Phytochemistry and Healthy Foods Laboratory, Department of Food Science and Technology, Spanish National Research Council for Scientific Research (CEBAS-CSIC), Murcia 30100, Spain; avictorio@cebas.csic.es

**Keywords:** *Brassicaceae*, ingredients, phytochemicals, functionality

## Abstract

*Brassicaceae* vegetables are important crops consumed worldwide due to their unique flavor, and for their broadly recognized functional properties, which are directly related to their phytochemical composition. Isothiocyanates (ITC) are the most characteristic compounds, considered responsible for their pungent taste. Besides ITC, these vegetables are also rich in carotenoids, phenolics, minerals, and vitamins. Consequently, Brassica’s phytochemical profile makes them an ideal natural source for improving the nutritional quality of manufactured foods. In this sense, the inclusion of functional ingredients into food matrices are of growing interest. In the present work, *Brassicaceae* ingredients, functionality, and future perspectives are reviewed.

## 1. *Brassicaceae* Family: A Rich Mine of Bioactive Phytochemicals

*Brassicaceae* family vegetables have an ample worldwide distribution, which can be found in all continents except Antarctica [1,2,3,4]. One of the most striking features of this botanical family is the presence of several kinds of secondary metabolites with a distinctive taste, and also interesting bioactivities. The most deeply studied are the glucosinolates (GSL) and their breakdown products, isothiocyanates and indoles [5,6,7]. Moreover, these species are also rich and possess unique profiles of phenolic compounds, carotenoids, and other groups of less studied compounds such as phytoalexins, terpenes, phytosteroids, and tocopherols, here reviewed.

### 1.1. Phenolic Compounds

Phenolic compounds are a large class of plant secondary metabolites characterized by having at least one aromatic ring with one or more hydroxyl groups attached, showing a diversity of structures, ranging from rather simple and low molecular weight structures to complex polymeric compounds. More than 8000 phenolic compounds have been reported on plant kingdom [8]. Phenolic compounds are very important regarding the quality of plant-based foods since they are involved in flavor features (e.g., astringency), and they are responsible for the color of some fruits and vegetables and also because they serve as substrates for enzymatic deterioration [9,10]. Finally, phenolic compounds are considered to contribute to the health benefits associated with dietary consumption of *Brassicaceae* species such as antioxidant capacity, anticarcinogenic power, anti-aggregation activity, activation of detoxification enzymes, among others (*Brassicaceae* bioactive properties are reviewed and discussed in Section 3 of the present study). The most important phenolic compounds present in *Brassicacea* family vegetables are the flavonoids and the hydroxycinnamic acids [8]. Among flavonoids, the most important group corresponds to the flavonols. Quercetin, kaempferol, isorhamnetin, and cyanidin are the most representative flavonols in these species, but their qualitative and quantitative profiles vary significantly among species. For example, cauliflower main phenolic compounds are quercetin aglycon and catechins, but in white cabbage, the main compound are kaempferol glucosides and epicatechins [11,12]. The phenolic profile can also vary within the same plant species according to the plant organ being studied; for example, cruciferous sprouts can contain from 2 to 10 times more phenolic compound when compared with roots and inflorescences, which are the most common plant organ consumed [13]. Recently, Fusari et al. (2019) reported for the first time important levels of *t*-resveratrol-*a p*-terostilbene phenolic compound extensively reported as a potent antioxidant molecule- in several members of this botanical family, reaching, for example, 84 µg/g dry weight level in rocket leaves (*Eruca sativa* L.) [12,14]. 

**Figure 1:**
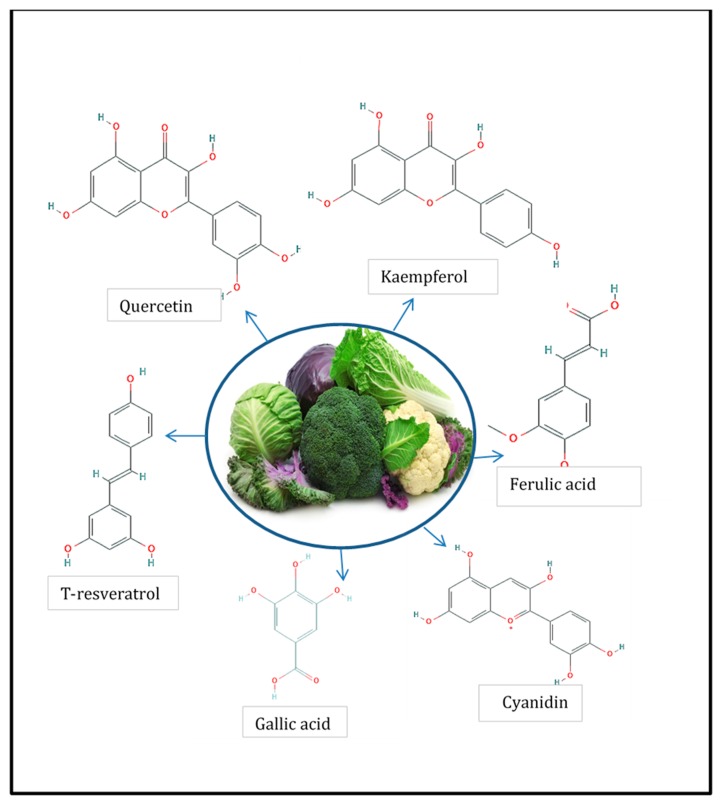
Phenolic compounds present in members of *Brassicaceae* [12].

Another group of phenolic compounds frequently detected in *Brassicaceae* vegetables is the hydroxycinnamic acid group, which is characterized by the C6–C3 structure and can be found free or conjugated with sugars or with other acids. The most common in this species are ferulic acid, sinapic acid, caffeic acid, and *p*-coumaric acid, but as occur with flavonols, this varies significantly according to the plant species considered. For example, sinapic acid is the main hydroxycinnamic acid present in rocket salad but is absent in red cherry and daikon radishes [11]. Another example of this considerable variation among species is ferulic acid, which represents the main hydroxycinnamic acid for red cabbage but is absent in radish and rocket leaves [12]. In Figure 1, the chemical structure of the phenolic compounds usually present in *Brassicaceae* is shown.

Anthocyanins have also been reported in *Brassicaceae* vegetables. For example, the red or violet pigmentation of red cabbage, purple cauliflower, or red radishes is caused by anthocyanins. The major type of anthocyanins differs among species. While red radish contains mainly cyanidin and peonidin anthocyanins aclylated with aromatic acids [15], red cabbage and broccoli sprouts contain mainly cyaniding glucosides derivatives [16]. Besides, Lo Scalzo et al. (2008) [17] found that the *p*-coumaryl and feruloyl esterified forms of cyanidin-3-sophoroside-5-glucoside were predominant in cauliflower, while the sinapyl one was mostly present in red cabbage. In another study, Otsuki (2002) [18] found 12 different anthocyanins in radish roots, 6 of them corresponding to the pelargonidin derivatives. In red cabbage cultivars, the predominant anthocyanins resulted in being nonacylated cyanidin- glucosides [19]. Altogether, these results indicate that anthocyanins, as with other phenolic compounds, vary according to the vegetable species, the plant organ, and the cultivars of the single species considered.

### 1.2. Organosulfur Compounds

Among the organosulfur compounds accumulated by cruciferous vegetables, glucosinolates (GSL)-sulfur-containing glycosides- are the main secondary metabolites found. Their presence is evidenced whenever its tissue is disrupted, and their breakdown products are the principal responsible for the sharp and bitter-tasting flavors of these vegetables. There have been described in plant kingdom more than 120 different GSL, but in *Brassicaceae*, the amount reaches around 96, of which some are unique of some specific gender or species [20]. While genetic factors determining mainly the type of GSL, environmental factors influence the amount of them. The induction of GSL following abiotic or biotic stresses has frequently been described in order to increase the phytochemicals levels when GSL are hydrolyzed by myrosinase (thioglucoside glucohydrolase, EC 3.2.1.147) upon tissue disruption, numerous breakdown products are formed, including isothiocyanates (ITCs), thiocyanates, nitriles, ascorbigen, indoles, oxazolidine-2-thiones, and epithioalkanes depending upon different factors like pH, temperature, presence of myrosinase-interacting protein, and availability of ferrous ion [21]. Among the different GLS-degradation products formed, the most abundantly formed at physiological pH are the ITCs, which are also considered the main responsible for cruciferous foods bioactivity. Since ITCs are very unstable, their health benefits depend on numerous variables related to several factors, such as the initial GSL concentration, cooking processes, amount of vegetable intake, and human metabolism. 

**Figure 2:**
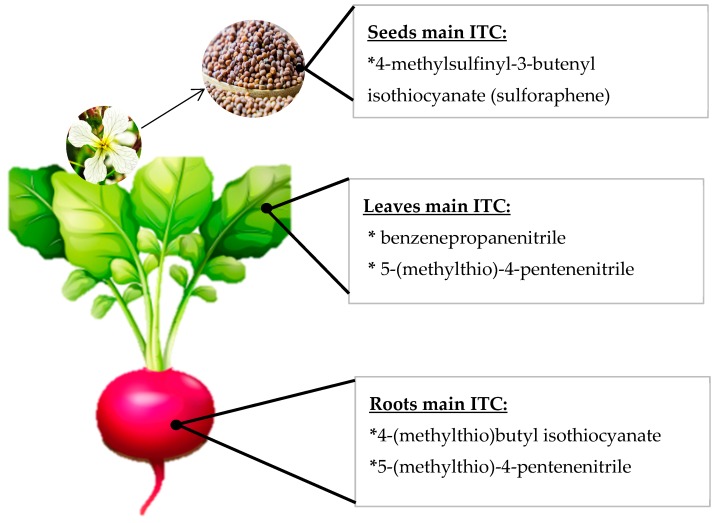
Schematic representation of different isothiocyanate (ITC) profiles in several radish plant organs.

Because the GSL profile varies between different species and the hydrolysis conditions determine the identity and amount of ITC compounds formed, it is possible to find in the literature many ITC profiles for each species, and these profiles do not always coincide with each other. ITC profiles for each cruciferous vegetable have been extensively reviewed [22,23,24,25], and the reported information highly variable according to the extraction. It is also important to keep in mind that the majority of GLS will not always give rise to its ITC profile. For example, in radish, it has consistently been reported that the majority GLS is glucoraphasatin; however, the raphasatin levels found when the ITC profile was studied, were negligible or non-quantifiable because of the extraction conditions and the hydrolysis affecting the results, especially if the medium is polar or aqueous [26]. Furthermore, the ITC profile of each cruciferous species varies according to the cultivar or variety considered and also according to the plant tissue found [27,28]. For example, in rocket leaves, the main ITC generally detected corresponds to sativin [29], but in rocket seeds, the main ITC detected have been erucin and sulforaphane (SFN) [30]. Accordingly, in radish seeds and sprouts, the main ITC detected was sulforaphene according to several reports [13,27,28,31] (Figure 2), but in radish roots, the chief ITC have been raphasatin and sulforaphene [27,32]. In Figure 2, a graphical example of the ITC variation inside a single plant is schematized. The foregoing demonstrates that, in order to inform the ITC profile of a cruciferous vegetable, it is very important to consider, in addition to the species, the cultivation, the plant organ under study, and the extraction and detection techniques in order to make inferences and comparisons among studies.

### 1.3. Carotenoids

Carotenoids are highly pigmented phytochemicals that possess a C40 backbone structure and are classified as symmetrical tetraterpenes. They are produced in many plants and microorganisms, and they can be yellow, orange, or red pigments. Some carotenoids such as β-carotene and α-carotene and β-cryptoxanthin have provitamin A activity since they act as precursors of vitamin A and, therefore, acquires an important function as a human health promoter. These compounds have been extensively studied for their health-enhancing properties and also for their biological functions as attractants to pollinators, as photoprotection pigments, and as light-harvesting pigments. Similarly, to phenolic compounds, the accumulation of carotenoids in cruciferous is regulated by the environment, tissue type, and developmental stage [33,34]. These groups of bioactive compounds have not been deeply characterized for *Brassicaceae* species, but it has been reported that the predominant ones are β-carotene and luteolin, but variable amounts of zeaxanthin, cryptoxanthin, neoxanthin, and violaxanthin have also been detected [35,36,37]. Other reports have found that carotenoid-containing cruciferous vegetables include kale (*Brassica oleracea* L. convar. Acephala var. sabellica), brussels sprouts, broccoli, cauliflower, red cabbage, white cabbage, pakchoi (Brassica rapa subsp. chinensis), and kohlrabi (Brassica oleracea var. ganglyoides) [38,39]. Kale is considered the richest cruciferous source of this compound, surpassing cabbage in about 40 times [35]. Among these, kale stands out for its high contents, not only within the cruciferous but also when compared to other vegetables, resulting in one of the main dietary source of carotenoids [35,40,41]. Kale main carotenoids have been proposed to be zeaxantin and luteolin [40] but important differences were found among several kale cultivars [42]. Carotenoid profile among *Brassicaceae* species varies greatly; for example, broccoli contains mainly β-carotene and luteolin [43], cabbage contains mainly luteolin, followed by β-carotene, zeaxantin, and α-carotene [44].

### 1.4. Other Terpenes Present in Brassicaceae Vegetables

Other naturally occurring terpenes compounds that can be found in *Brassicaceae* vegetables are tocotrienols and tocopherols. According to Podsedeck (2007) [45], the descending order of total tocopherols and tocotrienol content in Brassica vegetables is as follows: broccoli > broccoli sprouts > cabbage. Furtheremore, Kurilich et al. (1999) [46] reported that kale was the best source among other *Brassicaceae* vegetables of α-tocopherol and γ-tocopherol. Beside *Brassicaceae* vegetables, these compounds have also been reported in oils and cereals [47]. These phytochemicals have been extensively researched due to its anticarcinogenic properties [48,49]. Another bioactivity extensively reported in these lipid-soluble compounds is the antioxidant activity through hydrogen atom transference [50].

Phytosterols are another important terpene subclass. It has been reported to possess anti-inflammatory, anti-neoplastic, anti-pyretic, and immune-modulating activity. Also, it has been reported that phytosterols reduce serum or plasma total cholesterol and low-density lipoprotein (LDL) cholesterol [51]. Among cruciferous vegetables, Brassica napus L., known as rapeseed, is the most abundant natural source of phytosterols, reaching levels of up to 9.79 gr/kg oil [52]. Another rich source of phytosterols among cruciferous vegetables is Brassica Juncea, of which, according to the cultivar analyzed, different compositions and levels of phytosterol can be detected [53].

### 1.5. Phytoalexins

These groups of compounds were described initially in 1940 and are considered phenolic-related compounds with highly diverse chemical structures and several bioactivities, including anti-cancer properties. They possess low molecular weight and are thought to serve as an important defense mechanism for the plant [54,55]. *Brassicaceae* members containing phytoalexins present an indolic ring with C3 substitutions with N and S atoms, which confers a unique structure among other vegetables. The proposed biosynthetic pathway for phytoalexins formation includes brassinin formations from GSL; thereafter, other related phytoalexins are produced from brassinin. Klein and Sattely (2017) [55] reported that over 30 compounds arise from oxidative tailoring and rearrangement of Brassinin. Among edible cruciferous, phytoalexins have been reported in *Brassica napus* [56], *Brassica oleracea* [57], *Brassica Juncea* [58], *Sinapsis alba* [59], *Wasabi japonica* [60], and in *Raphanus sativus* [61].

### 1.6. Alkaloids

Alkaloids are secondary metabolites of plants synthesized from amino acids. These nitrogen compounds have been reported in several *Brassicaceae* species, including *Capsella bursa pastoris, Lepidium cartilacineum, Nasturtium montanum,* and *Raphanus sativus,* among others [62]. Among edible *Brassicaceae* alkaloid compounds have also been reported in a cabbage cultivar collection [63], in cabbage seeds by Mohammed (2014) [64], in cauliflower leaves [65], in broccoli florets [66]. and red cabbage florets [67]. Besides, a screening of tropane alkaloids—a class of alkaloids typically found in *Solanaceae* vegetables—in 43 different *Brassicaceae* species reveal that 18 of them presented alkaloid of different structures, and the authors proposed that alkaloid compounds are typical secondary cruciferous metabolites [68].

As described above, the *Brassicacea* family is characterized by the presence of GSL and the isothiocyanates that are exclusive of this family. Also, *Brassicacea* stands out because they possess phytochemicals of multiple chemical groups, being these species’ excellent sources of bioactive compounds that have been studied throughout history. Currently, modern analytical techniques allow us to expand the knowledge about new compounds and metabolites. Consequently, it is possible to understand that each species, each Brassica-derived product, is themselves a mixture of multiple components. For this reason, an exhaustive determination of their phytochemical profiles must be made in each case.

## 2. “Functional” Foods Based on Brassicas: Concepts and Relevance for Development of New Products

### 2.1. Origin of the “Functional Food” Concept

The origin of the concept of functional food dates to 1980 with the introduction of the concept “FOSHU” (Foods for Specified Human Health) in Japan. This tag system pretended, for first time in the world, to regulate the employment of health claims in the market [69]. From this point, the regulation of functional foods has been in constant evolution in Europe by the EFSA (European Food Safety Authority), in the USA by the FDA (United States Food and Drug Administration), or in Canada by the CFIA (Health Canada’s Food Directorate and the Canadian Food Inspection Agency), among others [70,71,72]. Nowadays, different unofficial definitions and concepts of functional foods coexist, being dependent of diverse factors: the country of the origin, in the case of a food product; the main characteristics of the manufacturing and the main use of this product; the specific criteria of an author, in the case of an article [73]. In this sense, a global definition can be outlined, based on specific definitions collected in recent literature [74]: A functional food must have a nutritional function that contributes to nutritional benefit on the consumer health, besides have been subjected to a technological process, in order to add a beneficial ingredient or eliminate a harmful one. In addition, it is interesting to mention the concept “nutraceutical” (classified by the European Union like “dietary supplement) [75], a kind of functional product with a specific format similar to medicines (e.g., pills, tablets), like [76]. However, it would be a priority to clarify the limit between medicine and functional foods or nutraceuticals: it is possible to establish a mandatory common characteristic, and, without discussion, that is the preventive and non-resolving nature of functional foods against different diseases.

In general terms and due to the information exposed, it is feasible to establish the next division of this group of products:

- Functional foods with an added (or enhanced) ingredient that is associated with a health benefit. Example: milk chocolate enriched with kale [77].
- Functional foods without an ingredient (naturally present in the original product) with a health risk associated. Example: reduced fat/cholesterol mayonnaise [78]. Nutraceuticals example: microencapsulation of polyphenols extracted from red chicory and red cabbage [79]

Nowadays, consumers have introduced several modifications in their nutritional habits, due to the growing concern about health. This fact could explain the recent popularization of food products based on functional ingredients, including products based on Brassicas vegetables [80]. However, the introduction of new concepts like bioaccessibility (“digestibility”) and bioavailabity has caused a strong controversy due to the questionable functionality of these food products in scientific reality [81]. In fact, the responsible regulations of health claims demand studies in vivo to allow legal commercialization, in addition to official controls in vitro that guarantees the presence of the compounds, related to the health claim of the commercial product and the capacity of the organism to use such compounds. 

### 2.2. Functional Foods Based on Brassica Vegetables 

Dietary intake of Brassicas has shown a relevant influence in the control and incidence of diverse diseases like cancer, hypertension, diabetes, chronic inflammation, or oxidative stress, among others [82]. These benefits are associated to specific compounds with active properties over human health, such as polyphenols or GSL, previously described in Section 1. 

In this sense, fresh products such as broccoli or cabbage have reached a high production level nowadays, which leads to a high amount of by-products with interesting potential as biocompound sources. This valorization process is especially relevant in popular crops like broccoli, which represents 34% of total cruciferous production in the world, according to the Food and Agriculture Organization Corporate Statistical Database (FAOSTAT, 2017, www.fao.org/faostat/es/). Broccoli floret, the edible part, represents just 15% of the total vegetable, producing 85% (stems and leaves) of valuable by-products [83]. Other Brassica species by-products have been studied as sources of bioactive compounds, among them red radish or kale, among others [84]. 

Recent work has focused on the increase of the total quantity of bioactive compounds found in Brassica species by elicitation in order to enhance health benefits. In this way, biotic (plant hormones), and abiotic (e.g., LED light, temperature, humidity, irrigation) elicitors, have been applied to improve the quantity of polyphenols and GSL in broccoli sprouts (Brassica oleracea L. var. Italica), red radish sprouts (Raphanus sativus cv. Rambo), or chinese cabbage (Brassica rapa ssp. pekinensis), among others [85,86]. 

In light of improving the manufacturing of these functional foods, efforts have been focused, on one hand, in the optimization of isolation and elicitation of these bioactive compounds from Brassicas and, on the other hand, in the use of the by-products generated by the industry, to obtain socio-economically sustainable products [87,88,89].

## 3. Functionality: What Has Been Demonstrated and What Remains to Be Study

The development of functional foods or functional ingredients is possible thanks to the interaction of three actors: (1) consumers demanding healthy foods, (2) the industrial sector motivated to elaborate and labelling their food products with a functional claim, and (3) the scientific sector which is responsible to obtain the knowledge to support those claims. Scientific substantiations of claims are performed by taking into account the totality of the available pertinent scientific data and by weighing up the evidence. To support these claims, scientific evidence on functional assessment procedures is needed, as well as toxicological evaluations and standardize analytical methodologies for functional component quantification [90]. The European Commission Regalement indicates a ranking of tests that can be done to support health statements of certain products. These tests consider, among others, whether a specific effect attributed to the product is representative in a target population, and if the quantity of the food and pattern of consumption required to obtain the claimed effect could reasonably be achieved as part of a balanced diet. In order of decreasing preference, the "most reliable" would be the products that would have demonstrated their benefits in experimental trials in humans. For that reason, among all the available information on the functional properties of *Brassicaceae*, we consider in the first instance, those that involve the results of epidemiological studies. A bibliographic search was made with the keys “epidemiology” + “*Brassicaceae*” + “functional property” in the last ten years. It follows that from the spectrum of functional properties attributed to this botanical family, biological properties related to chemoprevention of cancer are the most evaluated (40%), followed very far by properties related to the prevention of cardiovascular diseases (6.6%) and antidiabetics (5.7%). These results allow us, a priori, to warn of the areas in which there is a shortage of epidemiological data.

In this regard, the extent of information supporting the benefits of consuming cruciferous vegetables is plenty and rather proven. However, when analyzing the functionality of *Brassicaceae*-based products, epidemiological results are scarce. The studies that can be found in this regard focus mainly on product development, and the study of in vitro properties associated with some bioactive compounds present in the final product. Alvarez-Jubete et al. (2013), for example, determined the glucosinolate and isothiocyanate contents as the antioxidant capacity of a Broccoli-based soup [91]. Other authors have evaluated different biological activities of *Brassicaceae* juices. Broccoli sprout juice has been studied as a potential therapeutic strategy for Alzheimer’s disease [92]. A broccoli–cabbage juice was also efficient as a lowering LDL-cholesterol agent [93]. The antibacterial properties against food-borne bacteria of *Brassica oleracea* juice have also been addressed [94]. Furthermore, Brussels sprouts juice affects the balance of colorectal cell proliferation and death in an animal model of colorectal neoplasia [95]. Even though these findings help to give birth to the idea that certain products and ingredients have potential functionality, clinical and epidemiological studies that yield more accurate findings regarding a real health-claim are still lacking. 

Unlike the above, there is plenty of epidemiological evidence indicating that cruciferous vegetable (CV) consumption has health-promoting effects on the consumers. Although *Brassicaceae* vegetables are associated with numerous biological properties, such as antioxidant, anti-inflammatory, anti-diabetic, neuroprotective, and cholesterol-lowering effects, the cancer-protective effects stand out from the other [93]. It has been proven that CV is protective against a range of cancers, GSL and their breakdown products being considered the biologically active constituents [96]. 

Meta-analysis are useful tools that allow statistical comparison of results among a large number of research articles that have common variables [97]. In other words, they serve to organize and simplify the findings concerning a specific subject, identifying broad trends and patterns. In this case, updated meta-analysis related to chemopreventive properties associated with CV intake were examined in order to get closer to valid health claims. Data on epidemiological studies were taken from an exhaustive review carried out using the most common and broad search engines available online up to 2010. The databases considered were SCOPUS, SCIENCE.GOV, and SCHOLAR GOOGLE. The current paper limits itself to an overview of epidemiological data on cancers with high incidence and mortality such as lung, colon, prostate, gastric, breast, and ovarium. Based on other reviews, like the one published by Van Poppel et al. (2000) [98], two types of epidemiological studies were considered. On one side, prospective cohort studies, in which diets of large groups of people are recorded, using surveys, and then, these people are controlled for disease occurrence. On the other hand, retrospective case–control studies were also considered. They are based on interviews or questionnaires to estimate dietary patterns in the near or distant past. Data from patients are then compared with data from disease-free controls. 

As a result of this search in the most important data bases regarding scientific reports and articles, using as key words the terms “meta-analysis isothiocyanates”, up to 15,000 results appeared in SCHOLAR GOOGLE, but fewer results appeared in SCOPUS and SCIENCE.GOV. It must be point out that not all these results are meta-analysis per se, given that the term “analysis” is also included in the search. Therefore, most results are related to in-vitro or in vivo studies, and not precisely epidemiological studies, like the ones we are interested in. Table 1 shows the most relevant up-dated meta-analysis carried out, considering CV or ITC intake.

Lung cancer derived from tobacco consumption is responsible for approximately 22% of cancer deaths [112]. Regarding this type of cancer, back in 2009, Lam et al. [111] started to systematize the available evidence through a meta-analysis based on epidemiological studies focusing on the potential gene–diet interaction between cruciferous vegetable intake and GSTM1 and GSTT1 (genes involved in detoxification). Back then, the evidence weakly showed an inverse association between lung cancer and CV consumption. Since then, dozens of case–control and cohort studies have been published. Liu et al. (2013) gathered this information to make an emphasis in lung cancer incidence in females, more specifically in Chinese women aged 40 to 70 years, through a prospective cohort study, which added to previous observational evidence up to 2011 [108]. In that occasion, it was observed that CV consumption might reduce the risk of lung cancer in women, especially in non-smoker ones. More recently, Zhang et al. (2018) concluded that CV intake was inversely associated with lung cancer risk. However, they recognized that current evidence is still limited and more short-term clinical Phase II and III trials are needed to elucidate further whether the inverse association reported for CV intake is due to ITC content or other bioactive compounds. 

On the other side, cancers related to colorectum, gastric, oesophagus, and oropharyngeal tissues constitute one of the most common causes of death from cancer throughout the world [112]. In regards to this type of cancers, a literature review and meta-analysis carried out by Johnson et al. (2018) [109] proved that higher consumption of CV might reduce the risk of colorectal and gastric cancers by approximately 8% and 19%, respectively. These protective effects in the colorectal tissues are hypothesized to be associated with genetic polymorphism of regulating genes of the glutathione S-transferase enzyme expression; however, evidence is inconclusive on this point. That is why further epidemiological studies with accurate dietary exposure measurements need to be done. A previous study carried out by Tse et al. (2012) also suggested the value of CV intake when considering colon preventive properties. In that meta-analysis, including 33 articles, results showed a statistically significant inverse association between CV intake and colon cancer. Broccoli, particularly, stood out from other species. 

Following this line of research, pancreatic cancer is responsible for 4.5% of the total number of deaths in 2018 [112]. In 2015, Li [103] showed that cruciferous plant consumption might be inversely associated with pancreatic cancer risk. Nevertheless, given that the number of studies included in this research is scarce, further evidence needs to be included in future meta-analyses. 

On another note, breast cancer is the second most common cancer diagnosed in the entire world. Given this high incidence, joint efforts are being made to prevent the development of this disease, and CV intake has been proven as a chemopreventive strategy against breast cancer cells. Gianfredi et al. (2017) [110] identified sulforaphane (SFN) and epigallocathechin gallate as modulators of epigenetic events in one breast cancer cell line, therefore interfering with tumor growth rate. The following year, Zhang et al. (2018) [107] conducted a case–control study among Chinese women, proving that CV providing GLS and ITC showed a significant statistically inverse association with breast cancer risk. Despite these promising results, future prospective epidemiological studies are needed to positively assess a breast cancer prevention claim concerning CV or CV ingredient consumption. Another woman-affecting disease is ovarian cancer. In regards to this ailment, Hu et al. (2015) [100] showed that CV intake was also effective to prevent this disease. 

Cancers affecting the urinary system are also affected by *Brassicaceae* vegetable consumption. Liu et al. (2013) [102] proved the consumption of these vegetables might decrease the risk of renal cancer, and in 2015, Veeranki and colleagues [104], concluded that ITCs have chemopreventive activities against bladder cancer. 

**Figure 3:**
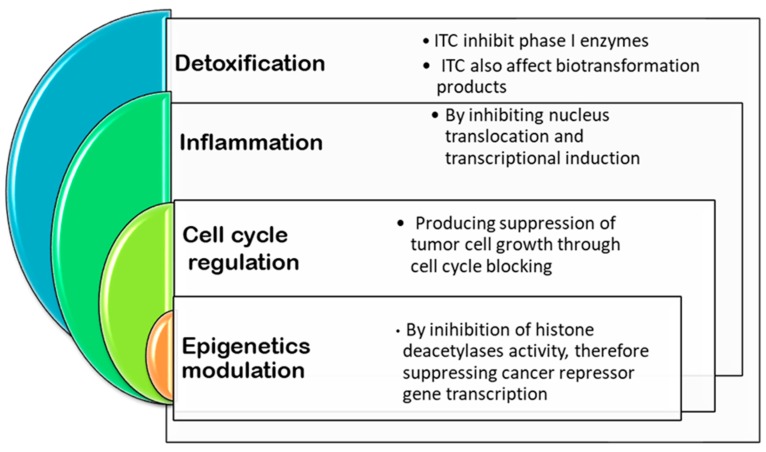
Mechanisms of action and signalling pathways implied for inhibiting carcinogenesis affected by isothiocyanates.

As can be appreciated, there are cumulatively epidemiological studies suggesting that cancer rates are associated with environmental factors, more precisely in this case with diet. Numerous researches have attempted to identify the dietary agents that may inhibit the multistage process of carcinogenesis [113]. In this sense, plenty of evidence regarding naturally occurring compounds that have shown cancer-preventive effects in experimental models is available. Particularly in *Brassicaceae* species, as several investigations show, isothiocyanates are the ones responsible for the chemopreventive activities [114]. There are other phytochemicals such as flavonoids present in Brassica species, that have cancer related properties; however, isothiocyanates are by far the ones with the greatest chemopreventive power [114]. Figure 3 shows the mechanism of actions during carcinogenesis process affected by ITCs.

Due to the wide range of GLS breakdown products that can be found in nature, it is not surprising that the number of cancer preventive mechanisms is so diverse. However, it must be noted that there is a dose-dependency in these responses generally; induction of cytoprotective genes and inhibition of CYP activity occurs at low compounds concentrations, whereas activation of cell cycle arrest and apoptosis occurs at higher levels of phytochemicals. Another major problem exists in interpreting experiments when using vegetable extracts because of their variable composition. Hence, uncertainty about attributing biological effects to specific phytochemicals exists. It is also important to consider the bioavailability of GLS breakdown products, which makes the interpretation of in vitro data more complex [115]. 

In summary, there is abundant scientific evidence that supports functional activities for the vegetables of the *Brassicaceas* family. However, the number of results related to *Brassicaceae* derived-products is lower. In agreement with point (2), each *Brassicaceae*-product (e.g., ingredient, phytotherapeutic, functional food) possesses a unique mixture of bioactive compounds that consequently evidences a spectrum of biological activities because of an additive/synergistic mechanism. From here arises the importance of studying each product particularly.

## 4. Food Products and Ingredients Enriched in Bioactives from *Brassicaceae*

The functional food market based on *Brassicaceae* vegetables is relatively recent. However, it contains interesting potential to offer new food products and formats with beneficial effects on health. In this sense, consumers, especially young adults and children, show some adversity in relation to the organoleptic characteristics, like the flavor of many cruciferous such as broccoli or radish [116]. For this reason, diverse new products based on functional ingredients from cruciferous families have been developed to facilitate the inclusion of this group of nutrients to the diet, into different and original formats (e.g., smoothies, soups, breads) [88]. In this way, the manufacturing of these new food products can include the use of by-products, or side-streams, to also reach a better environmental and socio-economic balance [117]. In addition, diverse nutraceuticals like broccoli pills, tablets, or powders have been commercialized to compensate for the absence of this group of vegetables in the diet [118]. 

Broccoli, cabbage, and kale are predominant in the search for new functional products based on Brassica vegetables (Table 2), due to their phytochemical composition and the extensive knowledge collected in the scientific literature until this moment. The processing of food to obtain the new functional products can cause diverse changes (advantageous or disadvantageous) into the food matrix. This is crucial for the design of novel food products. In this sense, it has been seen that the addition of broccoli to baked crackers improved the nutritional properties of the final product, whilst the addition of broccoli to a juice results in important losses of sulphoraphane (Table 2). On the other hand, the use of byproducts is promising, not only because of the use of non-marketable material but also for showing the advantages of using agro-waste instead of edible florets. 

Natural food matrix contents and bioactive compounds and grants the food the capacity to be adsorbed and metabolized by the organism [131]. However, the addition (or isolation) of some bioactive compound functional food products is not a guaranty of bioaccessibility and bioavailability, and more work is necessary to elucidate solutions against their insufficient presence in new products [132]. On the other hand, the food industry is trying to reduce the food processing to obtain products more similar to fresh-food: the concept called “minimally processed foods”. One example is broccoli hummus, which has shown promising nutritional values [133]. However, it is necessary to clearly establish the difference between fresh products and minimally processed foods to avoid the systematic and total consumption of fruits and vegetables instead off ready-to-eat fruits and salads, among other processed foods in the diet that, in addition, would result in an important impact on the environmental.

## 5. Food Products and Ingredients from *Brassica* spp.—Certain Commercialization Aspects

It is true that certain functional foods result in interesting physiological effects (Table 2), but it is necessary to stress that the novel developed “functional food” products cannot replace the nutritive qualities of fresh food [134]. Nevertheless, numerous food products are commercialized as “alternatives” for the consumption of *Brassica* vegetables, such as vegetable powdered formulas (broccoli https://www.bulkpowders.es/brocoli-en-polvo.html) or kale powder https://saludmediterranea.com/products/kale-col-rizada-en-polvo-salud-viva) in convenient but expensive formats (200 g >10 EUR/unit). We can ask ourselves if these are really necessary products, considering these prices. One kg of fresh broccoli (heads) is between EUR 1–2 in the supermarket, the sample place where these other products are sold in different shelves or areas of the store to highlight these added-valude products. Besides the price tag, the effects of industrial processing to obtain the products (e.g., powders) could degrade the phytochemical profile (and the label of these products sometimes is difficult to understand for the consumers) and the composition on the marketed products may be far distant from the natural content of the fresh produce for a given bioactive, and therefore, much less effective. 

Taking in consideration the mentioned situation, there is more interest so far in those foods that are minimally processed—once the organoleptic barrier is overpassed for the consumption of these smelly vegetable family—and ready-to-eat salads, sprouts and germinates of different cruciferous species are growing in demand and presence in the stores, even in countries that were not top consumers (such as USA or UK), as in the Mediterranean European countries [135,136], even in snack formats [137]. Therefore, there is a wide range of possibilities to exploit commercially.

When it comes to nutraceuticals or products alike, the situation is quite similar; their potential for specific population groups and pathophysiological situations where they can be used as coadjuvants is clear because of the isolation of the bioactive compounds in pharma-grade products such as pills, powders, capsules, but involving the elimination of the food matrix; in many circumstances, the isolated bioactive is not as bioavailable or metabolically active as in the natural food matrix [138].

The research in this area of functional foods and ingredients for new therapeutic applications keeps going further with the evaluation of functionalities in different chronic diseases; but many more studies will be needed to ascertain the “functionality” of these new products. For this reason, it is important to think about the population target these products are intended for: is the consumption of nutraceuticals for the general population really necessary or advisable? Will his trend lead consumers to avoid or dramatically reduce the consumption of fresh foods? If the current evidence from clinical studies and epidemiological data are still not totally clear or not totally acceptable for many of these new products, which are always much more expensive than fresh food, should we keep the wheel spinning and keep working hard on these products because of their (so far unclear) potential, or should we push for much more work from scientific research in collaboration with dietary and nutrition advice on eating a more sustainable, safe, and rich diet with plenty of “naturally functional” fresh foods (e.g., cruciferous sprouts, fresh foods enriched in bioactives) that would definitively contribute to wellbeing? Many open fronts remain waiting for answers in this global era of plants for food and health.

## Figures and Tables

**Table 1:** Latest most relevant evidence for the association of CV/ITC/GLS intake and cancer preventive effects.

Functional Property Addressed in the Meta-Analysis/Health Claim	Bioactive Compounds/Vegetables/Ingredients to Which the Bioactivity Is Attributed	Reference
Chemoprevention of melanoma	Isothiocyanates	[99]
May reduce ovarian cancer	Cruciferous vegetables	[100]
Protect against colon cancer	Cruciferous vegetables	[101]
May decrease risk of renal cancer	Cruciferous vegetables	[102]
Might be inversely associated with pancreatic cancer	Cruciferous vegetables	[103]
Chemoprevention activities against bladder cancer	Isothiocyanates	[104]
Inversely associated with type 2 diabetes	Cruciferous vegetables	[105]
Inversely associated with lung cancer	Cruciferous vegetables	[106]
Inversely associated with breast cancer	Glucosinolates (GSL) and isothiocyanates (ITC)	[107]
Decreased risk of renal cell carcinoma	Cruciferous vegetables	[102]
May reduce risk of developing lung cancer in females	Cruciferous vegetables	[108]
Decreased risk of developing colorectal and gastric cancer	Cruciferous vegetables	[109]
Chemoprevention of breast cancer	Sulforaphane (SFN)/Epicathechin Gallate	[110]
Weakly and inversely associated with lung cancer	Cruciferous vegetables	[111]

**Table 2:** Diverse experimental products based on functional ingredients from *Brassicaceae*.

Functional Food	Nutrients and Bioactive Compounds	Effects of Processing on Bioactive Compounds	References
Juice from Broccoli sprouts (*Brassica oleracea* L. var*. botrytis* subvar*. cymosa*)	SFN and Glucoraphanin (GRA)	Less amount of SFN present than expected from GRA dosage Lost of GLS/ITC during processing	[88]
Lentil flour fortified bread with addition of kale (*Brassica* *oleracea* var. *sabellica*) and pea leaves	Carotenoids, Chlorophylls Flavonoid glycosides Hydroxycinnamic acid derivatives	Less quantities of carotenoids and chlorophylls. Formation of derivatives (pheophytins) Losses of flavonoid glycosides and hydroxycinnamic acid derivatives	[119]
Muffins enriched with dietary fiber from kimchi byproducts	Dietary fiber	Enhanced of antioxidant capacity by adding kimchi fiber Decrease of color, height, and volume of the muffins Increased of hardness due to the weakening of gluten	[120]
Milk chocolate enriched with kale (*Brassica oleracea* var. *acephala)* and grapes	Phenolic compounds Dietary fiber Minerals	Some phenolic compounds transferred from kale or grapes to milk chocolate. However, the antiradical activity was not increased Enhanced total amount of fiber and minerals due to the addition of kale powder	[77]
Broccoli puree inoculated with lactic acid	Phenolic compounds GLS	Phytochemical total content was enhanced due to the fermentation of the lactic bacteria. The total content of sugar was lower than original broccoli puree	[121]
Broccoli puree inoculated with lactic acid	GRA SFN-nitrile	Improved stability of SFN Improved antioxidant capacity Preferential formation of SFN-nitrile (less potential as inducer of phase II detoxification enzymes than SFN) instead of SFN.	[122]
Broccoli soup with microalgae addition	Phenolic compounds	Improved antioxidant capacity due to the incorporation of the microalgae rich in bioaccessible phenolic compounds Preferential formation of SFN-nitrile instead of SFN.	[123]
Croquets with addition of red and green cabbage aqueous extract	Phenolic compounds	Improved antioxidant activity, better in croquets with green cabbage than croquets with red cabbage Organoleptic analysis indicates acceptability for consumers	[124]
“Kimchi” Prepared with Amtap Baechu Cabbage salted in Brine Solution	Gluconasturtin β-carotene, Pyropheophorbide A	Increase of the anticancer effect of “kimchi”	[125]
Baked crackers with addition of broccoli byproducts	Phenolic compounds Dietary fiber GLS	Improved antioxidant capacity Organoleptic properties unaffected by elaboration	[126]
Puree and juice made with Broccoli by-products (powder)	Epigallocatechin gallate	Improved antioxidant anticancer and anti-inflammatory activity by increased Epigallocatechin-gallate in puree Juices is not an optimal carrier of Epigallocatechin-gallate	[127]
Sponge cake with substitution of white flour (10% and 20%) by White Cabbage byproduct powder	Total dietary fiber	Increase of dietary fiber Decrease of total quantity of fat and carbohydrates Slight but acceptable decrease of organoleptic properties	[128]
Microencapsulation of polyphenols extracted from red chicory and red cabbage (nutraceutical)	Phenolic compounds	Stabilization of pH-dependent light-absorption properties of polyphenols Improvement of the thermal stability of polyphenols, mostly from red cabbage	[79]
Microencapsulated SFN from broccoli seed extracts (nutraceutical)	SFN	Powdered complex from Arabic gum and gelatin for encapsulating SFN from broccoli seeds.	[129]
Microencapsulated of Broccoli ingredient (nutraceutical)	Phenolic compounds Chlorophylls Carotenoids	Powdered complex obtained by coacervation for the stability of chlorophylls content Odor masking effects to improved acceptability for consumer	[130]

## Abbreviations

GSL	Glucosinolates
ITC	Isothiocyanates
CV	Cruciferous vegetables
SFN	Sulforaphane
GRA	Glucoraphanin
